# Infectious disease prediction model based on optimized deep learning algorithm

**DOI:** 10.3389/fpubh.2025.1703506

**Published:** 2026-01-14

**Authors:** Qian Cao, Junling Zheng, Yunyue Liu, Zongjin Li, Jianfei Xu, Huijuan Zhao, Lubin Feng, Yongchao Jin

**Affiliations:** 1 College of Science, North China University of Science and Technology, Tangshan, China; 2 School of Mathematics and Information Technology, Hebei Normal University of Science & Technology, Qinhuangdao, China; 3 Senior Department of Obstetrics and Gynecology, Chinese PLA Hospital, Beijing, China; 4 Periodical Press, North China University of Science and Technology, Tangshan, China; 5 School of Public Health, North China University of Science and Technology, Tangshan, China

**Keywords:** COVID-19, BiLSTM, GA, ARIMA, GA-BiLSTM-ARIMA

## Abstract

Since the end of 2019, a novel coronavirus known as COVID-19 has caused a severe outbreak worldwide. Due to the complexity of epidemic data, traditional algorithms have struggled to accurately predict the development of the pandemic. The Autoregressive Integrated Moving Average (ARIMA) model is capable of capturing time-based trends in epidemic data, including seasonality, cyclic patterns, and long-term trends, which helps improve the accuracy of forecasting future epidemic trajectories. The Bidirectional Long Short-Term Memory (BiLSTM) network, a variant of the Recurrent Neural Network (RNN), is highly effective in handling sequential data. In epidemic data analysis, BiLSTM models can be applied to forecast future trends or conduct time series predictions. BiLSTM is able to capture temporal relationships and sequential patterns within data, thereby providing more accurate predictions. Genetic Algorithms (GA), inspired by biological evolution through operations such as selection, crossover, and mutation, offer an efficient approach to identifying the best-fit models and parameter configurations. By using GA, we can iteratively optimize epidemic forecasting models and enhance their performance over time. In this study, we proposed a hybrid model called GA-BiLSTM-ARIMA. Using COVID-19 case data from Japan, we calculated the GA-BiLSTM-ARIMA model's evaluation metrics: *RMSE, MAE, MAPE*, and *R*^2^, which were 2,262.42, 1,672.07, 6.81, and 0.9764, respectively. The results demonstrate that the hybrid model outperforms both the standalone BiLSTM and ARIMA models in predictive performance. The GA-BiLSTM-ARIMA model successfully integrates the strengths of different models through a systematic and intelligently optimized hybrid strategy. When forecasting infectious disease time series data, this model achieves higher and more robust predictive accuracy compared to traditional single models or partial hybrid models. This type of analysis supports the development of more effective prevention and control strategies and delivers accurate information and early warnings to the public and policymakers, contributing to a better global response to pandemic challenges.

## 1 Introduction

Since 2019, the world has experienced a major outbreak of infectious disease caused by a novel coronavirus, first identified in Wuhan, Hubei Province, China. Through extensive scientific research and ongoing clinical analysis, experts have determined that the virus is highly contagious and capable of sustained human-to-human transmission. Due to its rapid spread and potential lethality, the virus quickly escalated into a global crisis. The COVID-19 pandemic has had a profound impact on the economic, social, and healthcare systems of countries worldwide. In an effort to contain the virus, many governments implemented strict lockdown measures, including travel restrictions, school and business closures, and mandatory home quarantines.

Extensive research on epidemic data prediction has been conducted both domestically and internationally. Numerous scholars and research teams have proposed a wide range of models and methodologies to forecast the development trends of epidemics. Raghvendra Jain employed generalized additive models (GAMs) to explore the relationship between predictors (with a 1-month lag) and clinical data for dengue hemorrhagic fever (DHF). He evaluated the predictive performance using Root Mean Square Error (RMSE), SRMSE, and adjusted R-squared values (1). Iman Rahimi provided a comprehensive review and analysis of key machine learning forecasting models applied to COVID-19. He emphasized the importance of scientometric and bibliometric analysis, discussing keyword trends, subject areas, model classification, evaluation criteria, and comparative methodologies. His study identified the most effective models used by researchers to predict the pandemic. Using publicly available data from Hubei Province, Cleo Anastassopoulou estimated key epidemiological parameters by calibrating a Susceptible-Infected-Recovered-Deceased (SIRD) model to the reported data. The model effectively forecasted the epidemic's progression (2). Henrique Mohallem Paiva proposed a dynamic model to describe and predict COVID-19 transmission, based on a framework previously used for the Middle East Respiratory Syndrome (MERS) outbreak. His model enables authorities to anticipate the evolution of the disease and plan appropriate interventions (3). Duccio Fanelli analyzed the temporal dynamics of the COVID-19 outbreaks in China, Italy, and France. By applying simple day-lag mapping and a modified SIRD model, he observed universal spreading patterns and accurately predicted the epidemic peak in Italy (4). K. C. Santosh explored various models including SEIR/SIR, agent-based models, and curve-fitting approaches. In addition, he highlighted the use of machine learning models built on statistical techniques to raise awareness among governments and citizens about potential outbreak consequences (5). Sheng Huaxiong applied the classical SIR model combined with a differential driving method to analyze and predict epidemic trends, demonstrating strong alignment between theoretical and observed values (6). Jin Yongchao proposed three hybrid forecasting models: CNN-LSTM-ARIMA, TCN-LSTM-ARIMA, and SSA-LSTM-ARIMA with the aim of improving the accuracy of COVID-19 pandemic predictions by integrating both linear and nonlinear components. Among these, the results demonstrated that the CNN-LSTM-ARIMA model achieved the highest predictive accuracy (7). Qu Zongxi proved that the government response index, strictness index, containment and health index and economic support index were significantly negatively correlated with the number of confirmed cases and deaths of new crown. On this basis, a multivariate long short-term memory network model was established to predict the confirmed cases and deaths of new crowns. The study found that the multivariate deep learning model with coupling variable correlation and lag was better than other comparison models (8).

However, the spread of COVID-19 is highly complex and involves a great deal of uncertainty. As a result, some predictions deviate significantly from actual values and fail to produce consistent outcomes. Forecasting epidemic data is an extremely challenging task, influenced by various factors such as policy interventions and population mobility. Therefore, accurate prediction of epidemic trends remains a complex issue that requires comprehensive consideration of multiple factors, along with continuous research and improvement.

Traditional ARIMA models have advantages in linear component modeling, while BiLSTM can effectively capture complex nonlinear features and contextual dependencies in sequences (9–11). However, a single model often has limitations, and the existing simple integration methods can not fully tap the potential of each model (12). To address this issue, this paper innovatively proposes a weighted hybrid model based on GA. Genetic algorithm (GA), as a global optimization algorithm, has unique advantages in finding the global optimal solution for complex problems, especially when the parameter space is large or traditional optimization algorithms cannot be used. Due to the strong nonlinearity and complexity of the time series data we process, genetic algorithms can avoid the problem of local optima and provide a global search framework (25). The core of this method lies in utilizing the global optimization capability of GA to adaptively find the optimal fusion weight for the prediction results of ARIMA and BiLSTM models. This strategy can theoretically achieve a dynamic optimal combination of linear laws and nonlinear patterns based on the predictive performance of each model.

**Figure 1:**
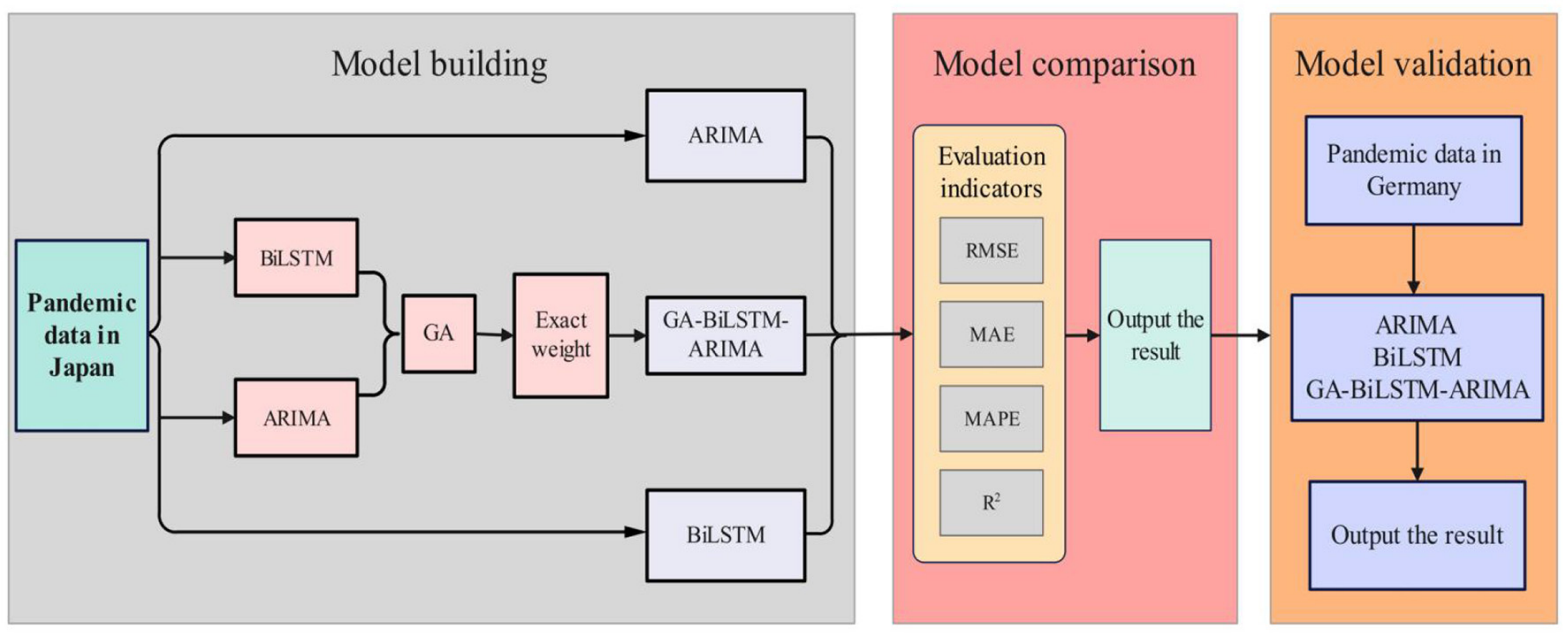
Technology roadmap.

In this study, the ARIMA model and BiLSTM model are first applied separately to predict epidemic data. Then, a genetic algorithm (GA) is used to combine the ARIMA and BiLSTM models by calculating the optimal weights for both. This hybrid model is referred to as GA-BiLSTM-ARIMA. The ARIMA, BiLSTM, and GA-BiLSTM-ARIMA models are used to forecast COVID-19 data in Japan, and their predictive accuracy is compared using three evaluation metrics: RMSE, Mean Absolute Error (MAE), and Mean Absolute Percentage Error (MAPE). Finally, the model's effectiveness is validated using COVID-19 data from Germany. The overall technical roadmap of this study is illustrated in Figure 1.

## 2 Materials and methods

### 2.1 Model selection

#### 2.1.1 BiLSTM model

The Bidirectional Long Short-Term Memory (BiLSTM) neural network is a specialized variant of the Recurrent Neural Network (RNN). Traditional RNNs often suffer from issues such as gradient explosion or vanishing gradients during training, which limits their ability to process long sequences and extract meaningful information from distant time steps. LSTM was introduced to address these limitations by incorporating gating mechanisms—namely input, forget, and output gates—that help regulate the flow of information and maintain long-term dependencies within the data (13).

BiLSTM builds upon the standard LSTM architecture by introducing two layers: a forward (or vertical) layer and a backward (or reverse) layer. The forward layer processes the input sequence in chronological order, while the backward layer processes it in reverse. This dual-layer structure enables BiLSTM to capture both past and future context simultaneously, leading to a more comprehensive understanding of sequence dependencies. The outputs from the forward and backward layers can be concatenated or otherwise combined to generate the final output (14).

Similar to standard LSTM, each BiLSTM unit consists of memory cells and gating mechanisms that regulate information flow. The key distinction is that BiLSTM processes the input sequence in both forward and backward directions. This allows the network to capture dependencies not only from past inputs but also from future inputs, enhancing its ability to model temporal relationships effectively (15).

*Step 1. Forget Gate Calculation*. The forget gate determines which information from the previous cell state should be discarded. It does so by taking the current input and the previous hidden state as inputs and computing a value between 0 and 1 for each element in the cell state, where 0 represents complete forgetting and 1 represents complete retention.


$$\begin{array}{l} f_{t}=\sigma \left(W_{f}\cdot \left[h_{t-1},x_{t}\right]+b_{f}\right) \end{array}$$ (1)

Among these variables, *h*_*t*−1_ represents the hidden state (output) from the previous time step, *x*_*t*_ is the input at the current time step, *W*_*f*_ and *b*_*f*_ are the weight matrix and bias term associated with the forget gate, and σ denotes the sigmoid activation function (16).

*Step 2. Input Gate Calculation*. The input gate determines how much of the new input should be added to the current cell state. It is computed using the current input *x*_*t*_ and the previous hidden state *h*_*t*−1_. The output of the input gate controls the extent to which new information is allowed to enter the memory cell (17).


$$\begin{array}{l} i_{t}=\sigma \left(W_{i}\cdot \left[h_{t-1},x_{t}\right]+b_{i}\right) \end{array}$$ (2)

*Step3. Candidate Cell State Calculation*. The candidate cell state ${\tilde{C}}_{t}$ is computed using the current input and the previous time step's output to help update the overall cell state. The formula is given by:


$$\begin{array}{l} {\tilde{C}}_{t}=tanh\left(W_{C}\cdot \left[h_{t-1},x_{t}\right]+b_{C}\right) \end{array}$$ (3)

In this equation, *W*_*C*_ is the weight matrix associated with the cell state, and *b*_*C*_ is the corresponding bias vector. Here, *h*_*t*−1_ is the hidden state from the previous time step, and *x*_*t*_ is the current input. The term [*h*_*t*−1_, *x*_*t*_] represents the concatenation of the two vectors along the column dimension. Multiplying this concatenated vector by the weight matrix *W*_*C*_and adding the bias *b*_*C*_ yields a new vector, which is then passed through a hyperbolic tangent (tanh) activation function to produce the candidate cell state ${\tilde{C}}_{t}$ (18, 19).

*Step 4. Cell State Update*. The current cell state *C*_*t*_ is updated by combining the previous cell state and the candidate cell state, weighted by the outputs of the forget gate and input gate, respectively:


$$\begin{array}{l} C_{t}=f_{t}*C_{t-1}+i_{t}*{\tilde{C}}_{t} \end{array}$$ (4)

In this equation, *f*_*t*_ is the forget gate output, *C*_*t*−1_ is the previous cell state, *i*_*t*_ is the input gate output, and ${\tilde{C}}_{t}$ is the candidate cell state output.

*Step 5. Output Gate Calculation*. The output gate determines how much of the cell state should be exposed as the hidden state output at the current time step. It is calculated as follows (18):


$$\begin{array}{l} o_{t}=\sigma \left(W_{o}\cdot \left[h_{t-1},x_{t}\right]+b_{o}\right) \end{array}$$ (5)

Here, *W*_*o*_ and *b*_*o*_ are the weight matrix and bias vector of the output gate, respectively.

*Step 6. Final Output Calculation*. The final hidden state *h*_*t*_ is generated by applying the tanh function to the updated cell state *C*_*t*_ and then multiplying it element-wise with the output gate (20):


$$\begin{array}{l} h_{t}=o_{t}⊙tanh\left(C_{t}\right) \end{array}$$ (6)

This output *h*_*t*_ represents the hidden state at the current time step and is passed to the next unit in the sequence.

**Figure 2:**
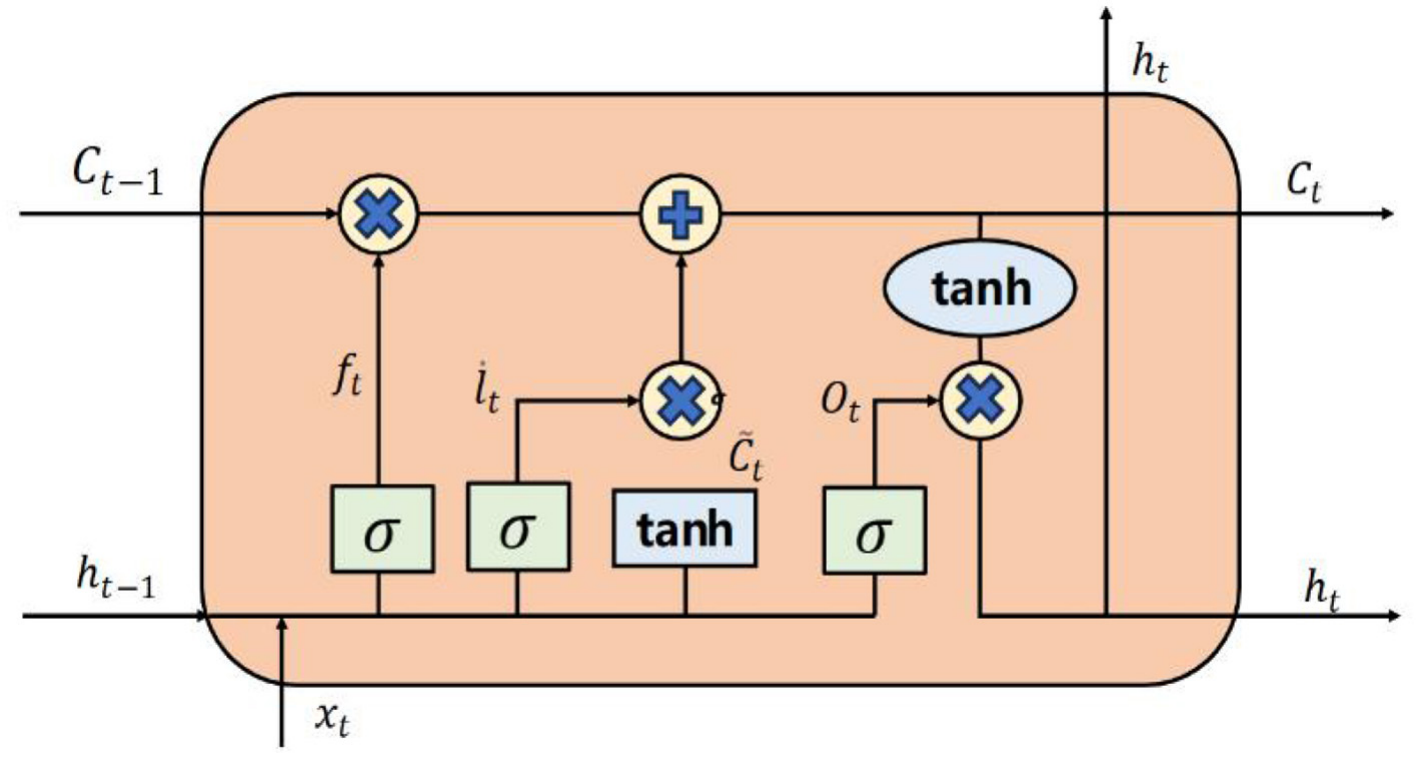
LSTM structural diagram.

The overall structure of the LSTM unit is illustrated in Figure 2.

**Figure 3:**
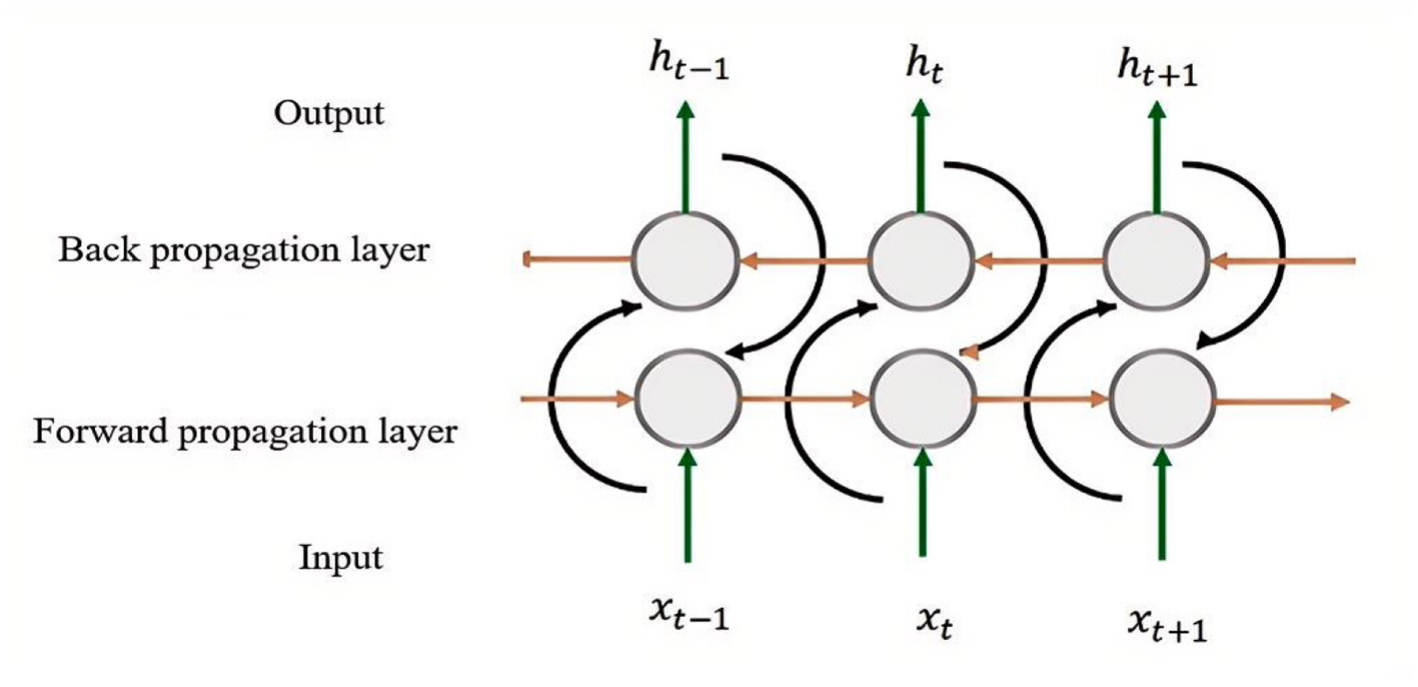
BiLSTM structural diagram.

The Bidirectional LSTM (BiLSTM) is an enhancement of the traditional LSTM architecture, incorporating two processing layers: a forward layer and a backward (reverse) layer. The forward layer processes the input sequence in chronological order, while the backward layer processes it in reverse chronological order. This dual-layer structure allows the BiLSTM to simultaneously capture contextual information from both past and future directions, enabling it to better model dependencies within the sequence data. The outputs from the forward and backward layers are then combined—typically through concatenation or other merging strategies—to produce the final output, as illustrated in Figure 3.

The computational process of the BiLSTM can be expressed as follows (25):


$$\begin{array}{l} \vec{h_{t}}=LSTM\left(\vec{x_{t}},\vec{h_{t-1}}\right) \end{array}$$ (7)

$$\begin{array}{l} \overset{⃖}{h_{t}}=LSTM\left(\overset{⃖}{x_{t}},\overset{⃖}{h_{t+1}}\right) \end{array}$$ (8)

Here, $\vec{x_{t}}$ and $\overset{⃖}{x_{t}}$represent the inputs at time step t or the forward and backward layers, respectively. Similarly, $\vec{h_{t-1}}$ and $\overset{⃖}{h_{t+1}}$ denote the hidden states from the previous time step in the forward direction and the subsequent time step in the backward direction. The final output *h*_*t*_ is obtained by merging the outputs from both directions, typically through concatenation (21):


$$\begin{array}{l} h_{t}=\left[\vec{h_{t}};\overset{⃖}{h_{t}}\right] \end{array}$$ (9)

This combined representation allows the BiLSTM to effectively capture both past and future context within the sequence data.

#### 2.1.2 ARIMA model

The ARIMA (Autoregressive Integrated Moving Average) model is a widely used statistical approach for analyzing and forecasting time series data. It captures underlying trends and seasonal variations to produce reliable predictions. The ARIMA model consists of three core components: the autoregressive (AR) part, the differencing (I) part, and the moving average (MA) part.

*Step1. Autoregressive (AR) Component*. The AR component represents the relationship between the current value and its previous values in the time series. The formula for the AR part is as follows (22–24):


$$\begin{array}{l} X_{t}=c+\phi_{1}X_{t-1}+\phi_{2}X_{t-2}+\cdots +\phi_{p}X_{t-p}+\varepsilon_{t} \end{array}$$ (10)

Where *X*_*t*_ is the value at the current time step, *c* is a constant, ϕ_1_, ϕ_2_, …, ϕ_*p*_ are the autoregressive coefficients, and ε_*t*_ is the white noise error term.

*Step2. Differencing (I) Component*. The differencing component is used to transform a non-stationary time series into a stationary one by removing trends and seasonality. It is typically done by subtracting the previous value from the current value:


$$\begin{array}{l} \Delta X_{t}=X_{t}-X_{t-1} \end{array}$$ (11)

Here, Δ*X*_*t*_ denotes the differenced time series.

*Step3. Moving Average (MA) Component*. The MA component models the dependency between the current value and past forecast errors. Its formula is as follows:


$$\begin{array}{l} X_{t}=\mu +\varepsilon_{t}+\theta_{1}\varepsilon_{t-1}+\theta_{2}\varepsilon_{t-2}+\cdots +\theta_{q}\varepsilon_{t-q} \end{array}$$ (12)

Where μ represents the mean value, ε_*t*_represents the error of the current moment, θ_1_, θ_2_, …, θ_*q*_ represents the moving average coefficient, and ε_*t*−1_, ε_*t*−2_, …, ε_*t*−*q*_represents the errors of the previous moments.

Combining all three components, the general form of the ARIMA model is expressed as:


$$\begin{array}{l} X_{t}=c+\phi_{1}X_{t-1}+\phi_{2}X_{t-2}+\cdots +\phi_{p}X_{t-p}+\mu +\varepsilon_{t}+\theta_{1}\varepsilon_{t-1}\\ \;+\theta_{2}\varepsilon_{t-2}+\cdots +\theta_{q}\varepsilon_{t-q} \end{array}$$ (13)

The ARIMA model is characterized by three parameters:

*p*: the order of the autoregressive part

*d*: the degree of differencing

*q*: the order of the moving average part

These parameters can be determined using tools such as the autocorrelation function (ACF) and partial autocorrelation function (PACF) plots. Once the model structure is identified, parameter estimation is typically performed using methods such as least squares or maximum likelihood estimation, after which the model can be used for forecasting future values in the time series.

#### 2.1.3 Genetic Algorithm (GA) model

Genetic Algorithm (GA) is a search and optimization technique inspired by the principles of natural evolution. It simulates the biological processes of selection, crossover, and mutation to explore the solution space and find optimal or near-optimal solutions through the mechanism of natural selection (25). GA is widely appreciated for its adaptability, global search capability, and effectiveness in solving complex problems. However, it also has certain limitations, such as low computational efficiency and the potential to converge prematurely to local optima.

The computational steps of a typical GA are as follows (26, 27):

*Step1. Population Initialization*. A set of individuals is randomly generated to form the initial population:


$$\begin{array}{l} P=\left\{p_{1},p_{2},\ldots ,p_{n}\right\} \end{array}$$ (14)

where *p*_*i*_ represents the *i-*th individual in the population.

*Step2. Fitness Evaluation*. The fitness of each individual is assessed using a fitness function, which varies depending on the problem. This function could be the objective function value or a measure of deviation. For each individual *p*_*i*_, compute the fitness value Fit (*p*_*i*_).

*Step3. Selection*. Selection is performed based on the fitness values to determine which individuals will be used to generate the next generation. The selection probability for each individual is calculated using the formula:


$$\begin{array}{l} P_{select}\left(pi\right)=\frac{Fit\left(p_{i}\right)}{\Sigma \left(Fit\left(p_{j}\right)\right)} \end{array}$$ (15)

where the denominator represents the total fitness of the population. Individuals with higher fitness have a greater chance of being selected as parents.

*Step4. Crossover*. Two parents, *p*_*i*_ and *p*_*j*_, are selected, and with a given crossover probability *p*_*c*_, a crossover operation is performed to generate two new offspring *c*_*i*_ and *c*_*j*_. Crossover methods include single-point, multi-point, and uniform crossover, among others.

*Step5. Mutation*. Each offspring *c*_*i*_ and *c*_*j*_ undergoes mutation with a certain mutation probability *p*_*m*_. The mutation introduces random alterations to maintain diversity in the population and explore new areas of the solution space.

*Step6. Population Update*. The current population is updated by combining the selected parents and the newly generated offspring to form the next generation.

*Step7. Termination Criteria*. The algorithm stops when a predefined termination condition is met (e.g., a maximum number of generations or a satisfactory fitness level). If not, the algorithm returns to step (2) and continues the evolution process.

### 2.2 Model evaluation metrics

Assess the accuracy of the model. This is crucial to the CD deployment and the various performance assessment criteria. We used MAE, MAPE, RMSE, and Coefficient of determination (*R*^2^).

The calculation formulas for four measures are as shown below (17).


$$\begin{array}{l} MAE=\frac{1}{m}\overset{m}{\underset{i=1}{\sum }}|{\hat{y}}_{i}-y_{i}| \end{array}$$ (16)

$$\begin{array}{l} MAPE=\frac{1}{n}\overset{n}{\underset{i=1}{\sum }}|\frac{{\hat{y}}_{i}-y_{i}}{y_{i}}| \end{array}$$ (17)

$$\begin{array}{l} RMSE=\sqrt{\frac{1}{m}\overset{m}{\underset{i=1}{\sum }}{\left({\hat{y}}_{i}-y_{i}\right)}^{2}} \end{array}$$ (18)

$$R^{2}=1-\frac{\sum_{i=1}^{m}{\left({\hat{y}}_{i}-y_{i}\right)}^{2}}{\sum_{i=1}^{m}{\left({\hat{y}}_{i}-y_{i}\right)}^{2}}$$ (19)

In the above formulas (16)–(19), n is the total sample size, ŷ_*i*_ is the predicted value of the model, *y*_*i*_ is the true value.

### 2.3 Dataset selection

**Figure 4:**
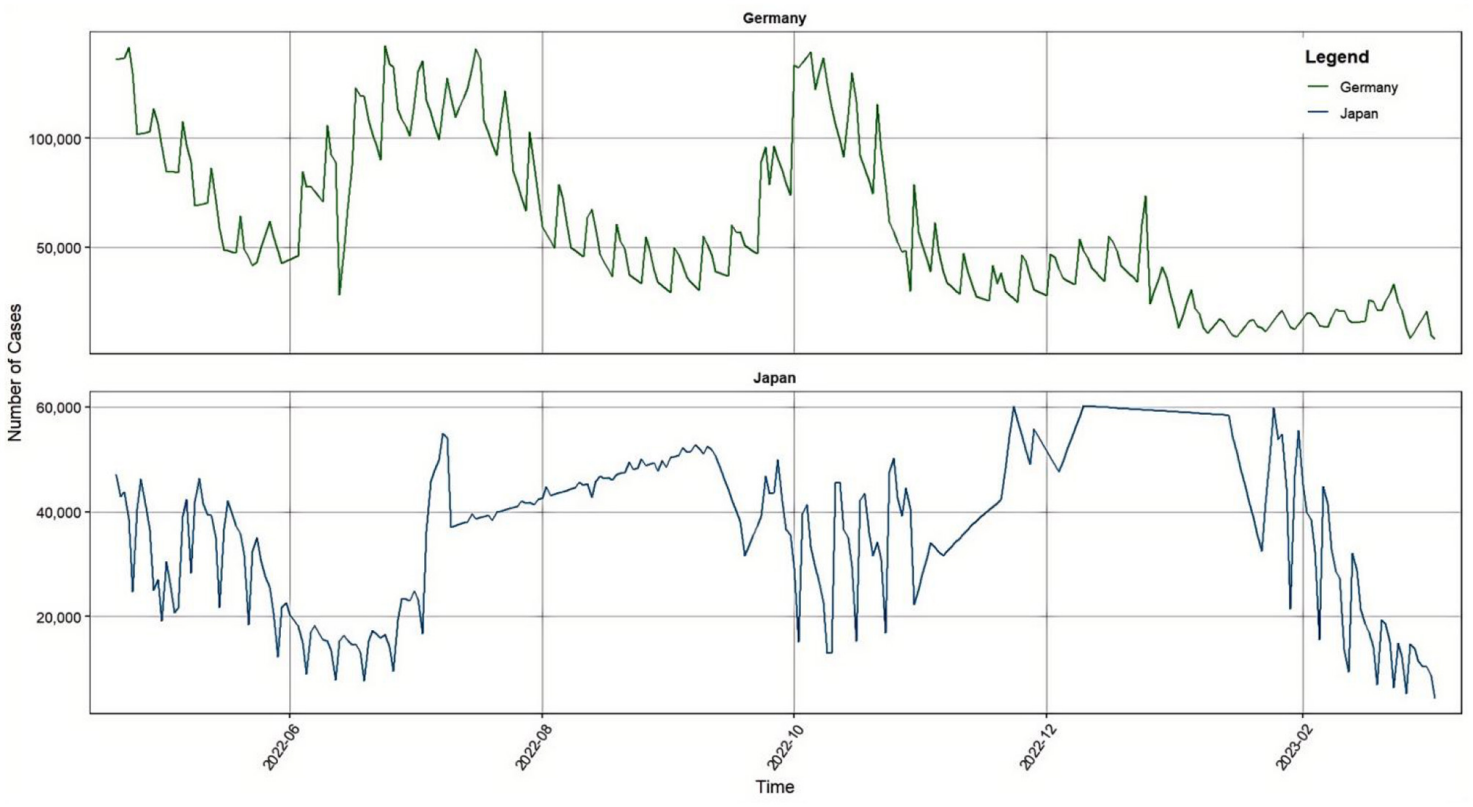
Confirmed data from Japan and Germany.

The data is sourced from the Johns Hopkins University website. In the data pre-processing stage, for the outliers in the time series, the sliding window method combined with Z-score is used to detect: the local mean and standard deviation are calculated with 10 days as the window, the data points that exceed the mean ± 3 times the standard deviation are marked, and the outliers are filled by linear interpolation. From descriptive statistics, the mean and standard deviation of Japanese data are 38176.26 and 14765.4, respectively, while the mean and standard deviation of German data are 59428.47 and 37089.27, respectively. Both countries' sequences exhibit non-stationary features with a multimodal structure (see Figure 4).

## 3 Results

**Figure 5:**
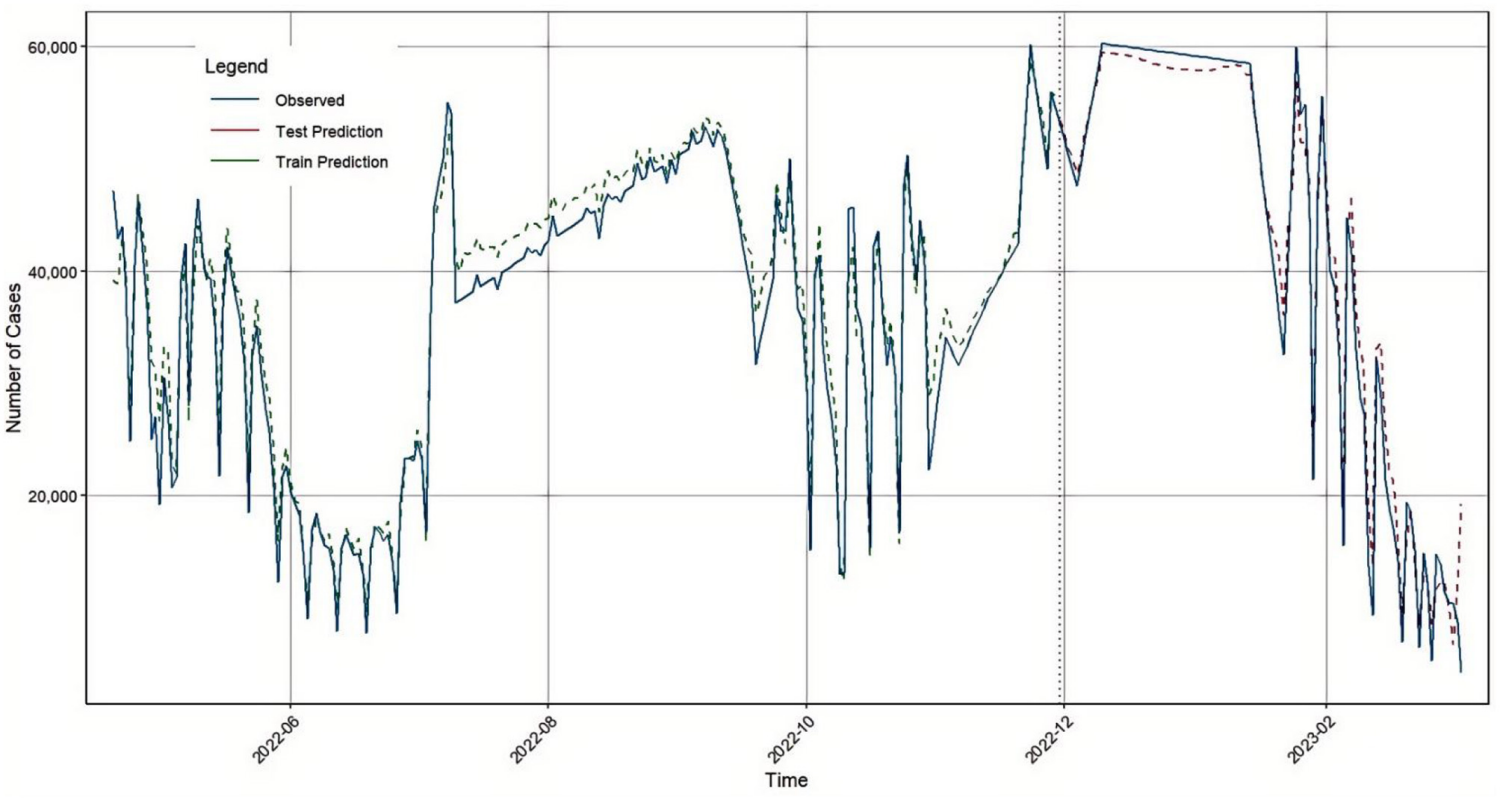
BiLSTM prediction results in Japan.

The BiLSTM model was developed using daily new confirmed COVID-19 cases in Japan, covering the period from April 20, 2022, to March 5, 2023. Both the input and output of the model consist of the number of new cases reported each day. The first two-thirds of the dataset were used as the training set to train the model, while the remaining one-third served as the test set to evaluate the model's generalization performance. The detailed parameter settings for the BiLSTM model are provided in Table 1. In rolling window cross validation, the key parameter settings are as follows: the training window size was set to 60% of the total data volume to ensure that each training cycle contains sufficient historical information to capture the dynamic features of the time series. The horizon was set to 30 time steps, reflecting the practical application requirements of the model in short—and medium-term prediction tasks. The window sliding step size was configured as 7 time steps, and the generalization ability of the model was systematically evaluated by sliding the window on the timeline in weeks. The detailed parameter settings for the BiLSTM model are provided in Table 1. Based on these parameters and the dataset, the BiLSTM model was constructed, and a time series plot comparing the predicted values with the actual values is presented in Figure 5. The performance evaluation metrics of the BiLSTM model on the test set are summarized in Table 2.

**Table 1:** BiLSTM model parameters.

**Parameter**	**Value**
Hidden units	300
Rate of learning	0.005
Optimization approach	Adam
Learn rate drop period	300
Learn rate drop factor	0.2

**Table 2:** Evaluation indicators of the BiLSTM model test set.

**Evaluation indicators**	**Numeric value**
RMSE	2405.98
MAE	1836.8
MAPE	7.39
R^2^	0.9734

**Figure 6:**
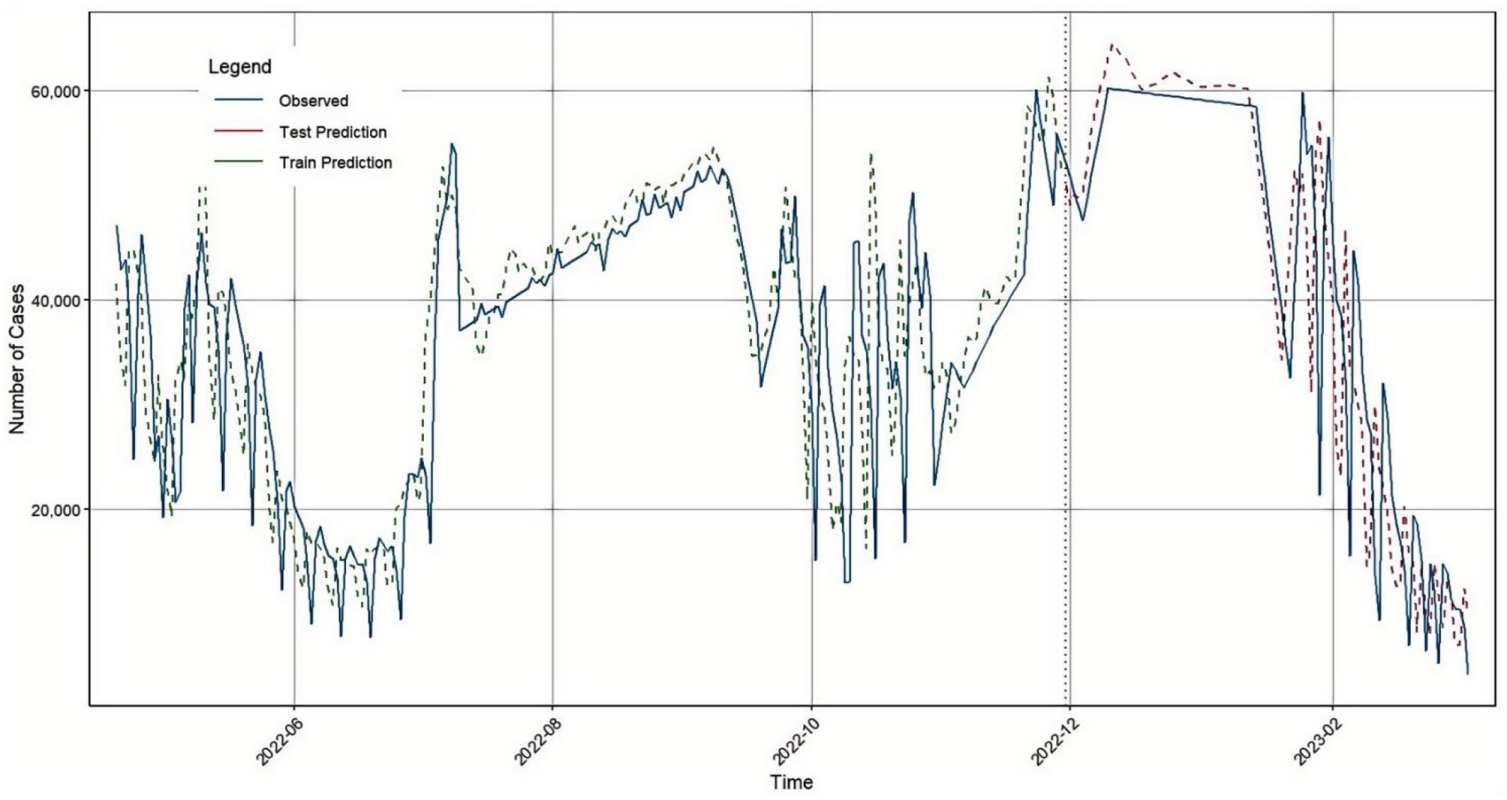
ARIMA prediction results in Japan.

The ARIMA model was constructed using daily new confirmed COVID-19 cases in Japan, spanning the period from April 20, 2022, to March 5, 2023. Initially, the original time series underwent first-order differencing to achieve stationarity. After differencing, the series became stationary and non-white noise. Based on the Bayesian Information Criterion (BIC), the optimal model was determined to be ARIMA (1, 1, 2), with white noise residuals, indicating a good fit. A time series comparison between the ARIMA model's fitted values and the actual values is shown in Figure 6.

The model's prediction performance on the test set is summarized in Table 3.

**Table 3:** Evaluation indicators of the ARIMA model test set.

**Evaluation indicators**	**Numeric value**
RMSE	7,385.03
MAE	5,156.67
MAPE	20.57
R^2^	0.7491

In this study, we combined the predictive outputs of both ARIMA and BiLSTM models using a genetic algorithm (GA), allowing the ensemble model to incorporate both linear and nonlinear components. The GA was applied by encoding two variables, *K*_1_and *K*_2_, as coefficients for the BiLSTM and ARIMA models, respectively. The objective was to minimize the root mean square error (RMSE) of the combined model. Through the GA processes of selection, crossover, and mutation, the optimal values *K1* = 0.9562 and *K2* = 0.0272 were obtained.


$$\begin{array}{l} \hat{y}=0.9562x_{BILSTM+}0.0272x_{ARIMA} \end{array}$$ (20)

The detailed parameter settings for the GA model are provided in Table 4.

**Table 4:** GA model parameters.

**Parameter**	**Value**
Population Size	50
Number of Generations	500
Crossover Probability (cxpb)	0.5
Mutation Probability (mutpb)	0.1

**Figure 7:**
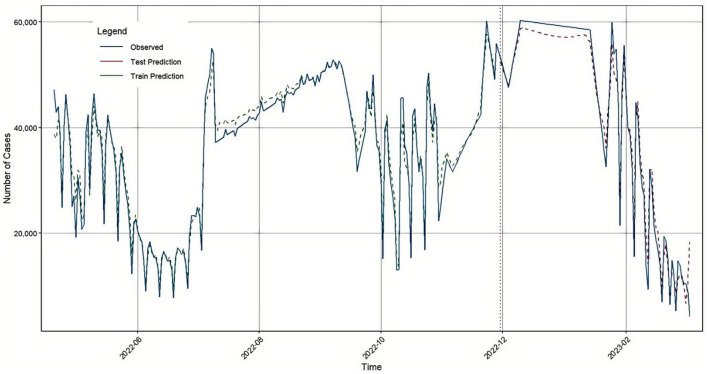
GA-BiLSTM-ARIMA prediction results in Japan.

As a result, the final forecast was generated based on the GA-BiLSTM-ARIMA model. A time series comparison of the model's predictions versus actual values is also presented in Figure 7, and the model's performance metrics are detailed in Table 5.

**Table 5:** Evaluation indicators of the GA-BiLSTM-ARIMA model test set.

**Evaluation indicators**	**Numeric value**
RMSE	2,262.42
MAE	1,672.07
MAPE	6.81
R^2^	0.9764

Compared with the individual ARIMA and BiLSTM models, the GA-BiLSTM-ARIMA model demonstrated superior predictive accuracy.

**Figure 8:**
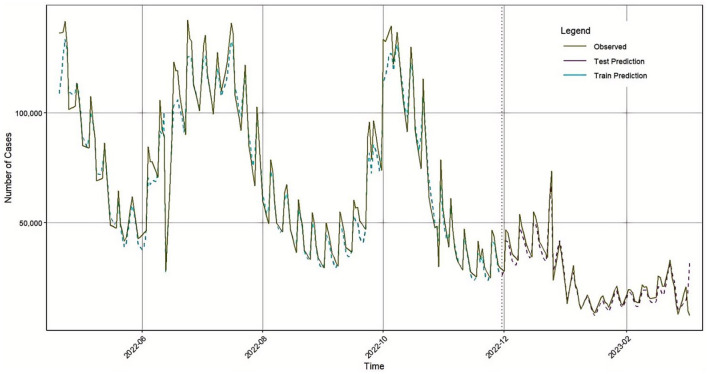
BiLSTM prediction results in Germany.

To validate the robustness and generalizability of the proposed hybrid model, the same modeling approach was applied to COVID-19 confirmed case data from Germany, covering the same time period (April 20, 2022, to March 5, 2023). The BiLSTM model parameters were kept consistent with those used in the Japan dataset. A comparison of the BiLSTM model's predicted values and the actual values for Germany is shown in Figure 8.

**Figure 9:**
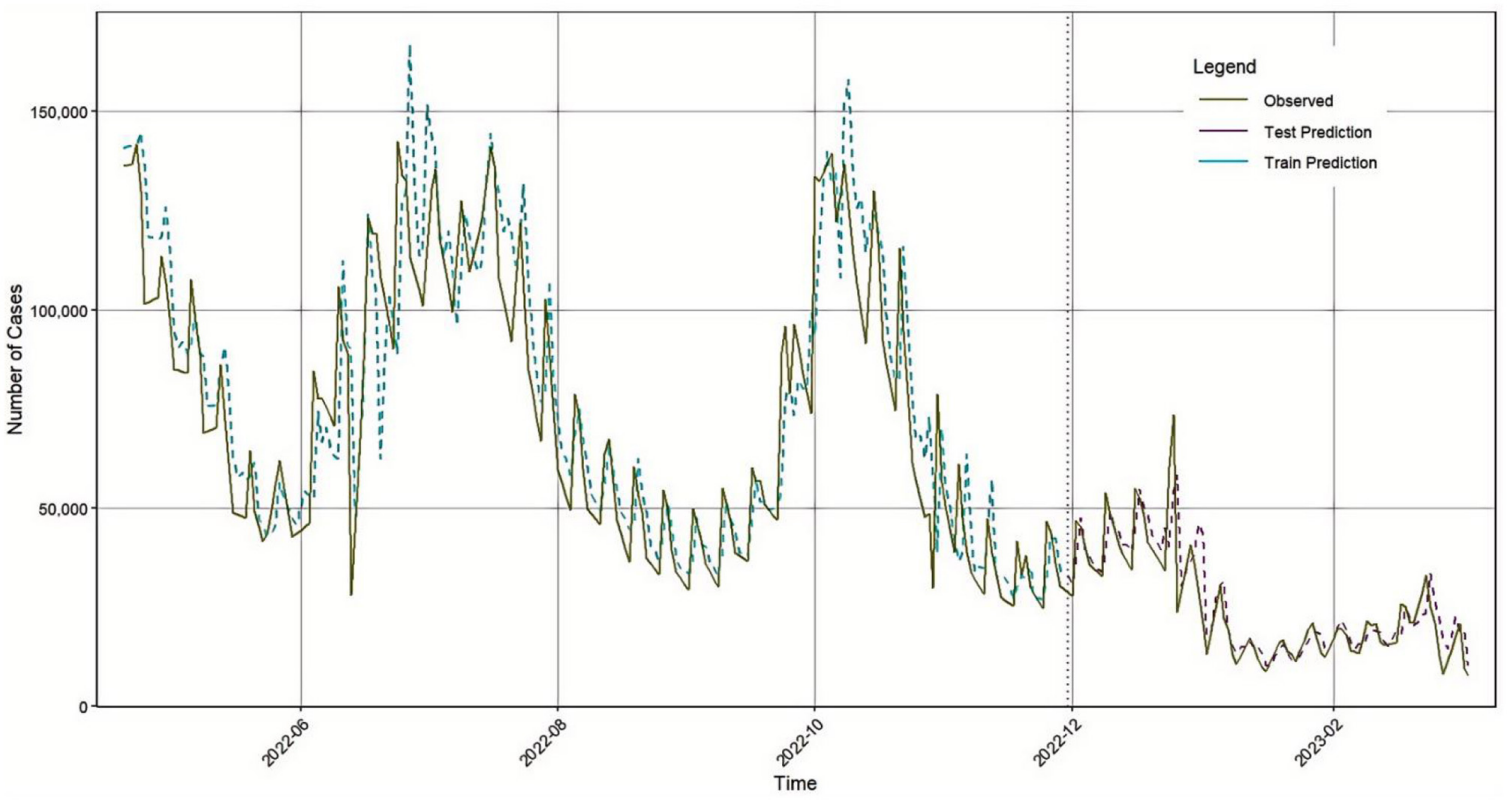
ARIMA prediction results in Germany.

After analysis, the ARIMA (1, 1, 1) model was confirmed as the optimal model. A time series comparison between the ARIMA model's fitted values and the actual values is shown in Figure 9.

Subsequently, the outputs of the BiLSTM and ARIMA models were weighted and optimized using a genetic algorithm, resulting in the following combined prediction formula:


$$\begin{array}{l} \hat{y}=0.918x_{BILSTM}+0.066x_{ARIMA} \end{array}$$ (21)

**Figure 10:**
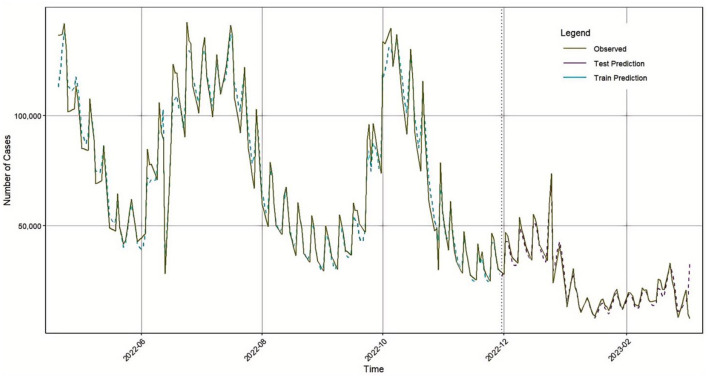
GA-BiLSTM-ARIMA prediction results in Germany.

Based on this formula, the final prediction results are illustrated in the time series plot shown in Figure 10.

To evaluate model performance, we calculated four common evaluation metrics: MSE, RMSE, MAE, and R^2^. The results are presented in Table 6.

**Table 6:** Evaluation indicators of the three models test set.

**Model**	**Evaluation indicators**	**Numeric value**
BiLSTM	RMSE	5,582.093
	MAE	3,976.007
	MAPE	8.487
	R^2^	0.9773
ARIMA	RMSE	13,133.822
	MAE	8,729.708
	MAPE	16.372
	R^2^	0.8472
GA-BILSTM-ARIMA	RMSE	5,278.33
	MAE	3,733.461
	MAPE	7.936
	R^2^	0.9797

These results clearly demonstrate the performance differences among the three models, with the GA-BiLSTM-ARIMA model achieving the highest predictive accuracy, outperforming both the standalone BiLSTM and ARIMA models.

## 4 Discussion

First, the BiLSTM model was used to predict the number of confirmed COVID-19 cases in Japan. The model's accuracy evaluation metrics: RMSE, MAE, MAPE, and R^2^ were 2405.98, 1836.8, 7.39, and 0.9734, respectively. Next, the traditional time series model ARIMA was applied to forecast the same dataset, yielding RMSE, MAE, MAPE, and R^2^ values of 7385.03, 5156.67, 20.57, and 0.7491, respectively. Considering that COVID-19 confirmed case data contain both linear and nonlinear patterns, we proposed a hybrid GA-BiLSTM-ARIMA model. This model uses a genetic algorithm (GA) to assign optimized weights to the BiLSTM and ARIMA models by multiplying them with their respective coefficients. The GA-BiLSTM-ARIMA combined model was then used to predict the confirmed COVID-19 cases in Japan, resulting in RMSE, MAE, MAPE, and R^2^ values of 2262.42, 1672.07, 6.81, and 0.9764, respectively. These results demonstrate that the GA-BiLSTM-ARIMA model significantly outperforms both the BiLSTM and ARIMA models in prediction accuracy.

To further validate the generalization ability and accuracy of the GA-BiLSTM-ARIMA model, all three models were applied to forecast the number of confirmed COVID-19 cases in Germany. The BiLSTM model achieved RMSE, MAE, MAPE, and R^2^ values of 5582.093, 3976.007, 8.487, and 0.9773, respectively. The ARIMA model produced RMSE, MAE, MAPE, and R^2^ values of 13133.822, 8729.708, 16.372, and 0.8472. The GA-BiLSTM-ARIMA model yielded improved metrics: RMSE of 5278.33, MAE of 3733.461, MAPE of 7.936, and R^2^ of 0.9797. The sequence of newly added cases in the epidemic often undergoes periodic outbreaks, declines, and re outbreaks, with significant structural changes and nonlinear fluctuations. ARIMA is based on linear autoregression and difference assumptions, and is more suitable for sequences that are approximately stationary or have strong linear dependencies; When the sequence exhibits strong nonlinearity, intra week effects, and sudden changes, its fitting and extrapolation abilities are limited. In contrast, BiLSTM is able to learn nonlinear mappings and capture long short-term dependencies without explicitly modeling stationarity conditions, thus performing better on this type of epidemic time series.

These results again confirm that the GA-BiLSTM-ARIMA hybrid model consistently outperforms the individual BiLSTM and ARIMA models in prediction accuracy. The GA-BiLSTM-ARIMA hybrid model proposed in this article demonstrates excellent performance in COVID-19 prediction, and its value lies not only in the improvement of prediction accuracy, but also in providing important technical support for public health decision-making. This model optimizes weight allocation through genetic algorithm, effectively integrating the linear capture ability of ARIMA and the nonlinear feature learning advantage of BiLSTM, achieving more reliable prediction of future epidemic trends.

## 5 Conclusions

The GA-BiLSTM-ARIMA model proposed in this study is a hybrid forecasting framework that integrates genetic algorithms with bidirectional long short-term memory (BiLSTM) networks and the ARIMA model. The genetic algorithm is used to optimize hyperparameters such as learning rate and hidden layer size through iterative selection and mutation, thereby enhancing the model's predictive accuracy. This hybrid model excels in processing sequential data, with performance further improved by the optimization capabilities of the genetic algorithm. Leveraging the powerful nonlinear fitting ability of BiLSTM networks, the model effectively captures complex features from input sequences—such as tidal levels influenced by nonlinear factors—and constructs an initial forecast. The residuals (error sequence) from the BiLSTM predictions are then modeled using ARIMA to capture underlying linear patterns. Finally, the predictions from the BiLSTM (nonlinear components) and ARIMA (linear components) are combined to produce the final forecast. Comprehensive evaluation using standard performance metrics demonstrates that the hybrid GA-BiLSTM-ARIMA model significantly outperforms individual models, offering superior accuracy and robustness in time series prediction.

In public health practice, this model has three core values. Firstly, its forward-looking predictions can provide critical warning window periods for disease control departments, supporting the development of early intervention measures. Secondly, accurate prediction of the growth trend of cases can guide the precise allocation of medical resources and the activation of emergency plans. Finally, the model can serve as a policy simulator to quantitatively evaluate the effectiveness of different intervention measures and assist the government in seeking the optimal balance between epidemic control and socio-economic operation.

Despite the excellent performance of the model, its performance is still constrained by data quality and timeliness, and key external variables such as virus mutations and changes in population immunity have not been explicitly included. The model only relies on daily newly confirmed cases as a single time series variable, ignoring multidimensional factors. In addition, the model assumes that linear and nonlinear features remain stable throughout the entire prediction period and cannot dynamically adapt to unexpected events such as virus mutations. Future research will construct a dynamic prediction framework for multi-source data fusion, which will be implemented through a “data layer model layer decision layer” approach. On the basis of adding new confirmed sequences, the data layer introduces process indicators such as genome monitoring, serological investigation and detection positive rate, hospitalization/ICU, etc., and unifies the time granularity through resampling and lag alignment to handle missing data and label uncertainty. The model layer adopts exogenous covariate multivariate prediction, and combines rolling updates and change point detection to trigger retraining to adapt to distribution drift. The output layer provides interval prediction, risk level and scenario prediction, providing a basis for resource allocation and intervention decisions, and enhancing robustness and transferability.

## Data Availability

The original contributions presented in the study are included in the article/supplementary material, further inquiries can be directed to the corresponding author.
